# Factors Affecting the Unconscious Bias of Healthcare Professionals in Obesity Care

**DOI:** 10.3390/jcm14051486

**Published:** 2025-02-23

**Authors:** Jana Makuc, Ana Ogrič Lapajne, Špela Hvalec, Mojca Jensterle, Andrej Janež

**Affiliations:** 1 Topolšica Hospital, Topolšica 64, 3326 Topolšica, Slovenia; 2 Faculty of Medicine, University of Ljubljana, Vrazov trg 2, 1000 Ljubljana, Slovenia; mojcajensterle@yahoo.com; 3 Community Health Centre Idrija, Otona Župančiča 3, 5280 Idrija, Slovenia; 4 Psychiatric Hospital Idrija, Pot sv. Antona 49, 5280 Idrija, Slovenia; spelahvalec@gmail.com; 5 Department of Endocrinology, Diabetes and Metabolic Diseases, University Medical Centre Ljubljana, Zaloška 7, 1000 Ljubljana, Slovenia; andrej.janez@kclj.si

**Keywords:** obesity care, bias, healthcare professionals

## Abstract

**Background/Objectives**: To assess the factors affecting the unconscious bias of healthcare professionals (HCPs) in obesity care. **Methods**: A cross-sectional, non-interventional, descriptive study collecting data via an online survey system was distributed via e-mail to 11,597 members of the Medical Chamber of Slovenia. Physicians were assigned into six HCP categories: (junior) resident physicians, primary care physicians (PCPs), internal medicine specialists, surgeons, dentists, and others. The online questionnaire was active for two weeks. **Results**: A total of 1248 physicians opened the survey link (10.8% response rate). Of the 898 physicians that engaged in the survey, 789 fully completed the questionnaire. Out of those physicians, 93.6% agreed that obesity is a disease, 83.7% were familiar with the definition, and 75.5% of HCPs were professionally interested in the subject. Overall, 39% of HCPs use the ICD obesity code, primarily primary care physicians and specialists in internal medicine. Notably, 82.0% of HCPs identified lifestyle change as the most effective intervention and believed that patients could lose weight with a serious attempt at a lifestyle change. This belief was particularly supported by male HCPs and HCPs under 40 years of age, who felt that patients were entirely responsible for their weight. The unconscious bias decreased with an increase in the HCPs’ body mass index (BMI), but at the same time, physicians with a higher BMI found obesity to be less important than other diseases (*p* = 0.036). Using composite answers, we found that the unconscious bias of HCPs toward obesity and effective obesity care was significantly related to gender (*p* = 0.017), age (*p* < 0.001), and BMI (*p* = 0.005), and was independent of an area of expertise. **Conclusions**: HCPs’ area of expertise impacted their professional standpoint (suggesting conscious bias), whereas male gender, a younger age, and a lower BMI affected unconscious bias toward obesity and its effective care. Despite their limitations, including the self-reported nature of the data, our findings can help to individualize educational strategies and create a more equitable environment in obesity healthcare.

## 1. Introduction

Obesity is a chronic, complex, and relapsing disease in which abnormal or excess fat has an impact on health, increases the risk of medical complications, and shortens life expectancy [1]. The prevalence of obesity is rising; according to the Slovenian National Institute of Public Health, the prevalence of overweight adult people (aged 25 to 74 years) in Slovenia was 39% in 2022, and 20% were living with obesity. Higher percentages were present in males, people with a lower educational level, and older adults [2]. World Health Organization data for 2022 shows an 18% prevalence of obesity and a 43% prevalence of overweight in adults older than 18 years [3].

Healthcare professionals (HCPs) should assess their attitudes and beliefs regarding obesity and consider how their attitudes and beliefs may influence care delivery [1]. The investigation of unconscious bias in healthcare, particularly in obesity care, is grounded in established cognitive and social psychological theories of bias formation and implicit attitudes. Several theoretical frameworks, such as the Implicit Bias Theory [4] and the Stereotype Content Model [5], highlight how subconscious associations influence clinical decision-making. The Dual Process Model of Bias [6] distinguishes between automatic (implicit) and controlled (explicit) cognitive processes, which may explain the divergence between HCPs’ professional perspectives and personal beliefs about obesity. HCPs should recognize that internalized weight bias in people living with obesity can affect behavioral and health outcomes. HCPs should avoid using judgmental words, images, and practices when working with patients living with obesity. They should avoid making assumptions that a patient’s ailment or complaint is related to their body weight [1].

The results of the ACTION-IO study showed that 68% of people with obesity and 88% of HCPs agreed with the statement that obesity is a chronic disease [7]. Despite recognizing obesity as a disease, most people living with obesity assumed full responsibility for weight loss (81%) [7]. Moreover, 30% of HCPs placed the responsibility for weight loss on people with obesity and 79% agreed that their patients would need to completely change their lifestyle in order to lose weight [7]. People with obesity are motivated to engage in weight loss efforts and would like their HCPs to initiate a conversation about weight. However, only 31% of HCPs thought that their patients were motivated to lose weight (as opposed to 48% of people with obesity confirming motivation) [7]. As obesity is a chronic disease, there is also a need to improve referrals and follow-up appointments for the management of obesity. Part of this effort calls for the eradication of the prevalent stigmatizing attitudes and for the eradication of HCPs’ misperception that people with obesity are not motivated [7]. Studies suggest that HCPs should strategically focus on reducing obesity bias and provide high-quality obesity management [8].

Although global studies have examined weight bias among physicians, the research gap in this area pertains to the lack of empirical data on how cultural, systemic, and professional factors shape bias within Slovenia’s healthcare settings. This study aims to bridge that gap by assessing both the explicit and implicit attitudes of Slovenian physicians towards obesity and its management, including the internalized acknowledgement of obesity as a disease and possible unconscious bias and stigma. The study’s secondary goals were to encourage HCPs’ internal reflection on the subject, especially regarding stigma and bias possibly affecting their care delivery.

## 2. Materials and Methods

### 2.1. Study Design, Participants, and Outcomes

We performed a cross-sectional, non-interventional, descriptive study collecting data via an online survey system (1ka, GO TEL, Ljubljana, Slovenia). The study was approved by the designated ethics committee (General Hospital Celje Ethics Committee; No. 58/2023/2, issued 5 May 2023) and nationwide distribution was further endorsed by The Medical Chamber of Slovenia’s Committee for Legal and Ethical issues (27 October 2023). Participant consent was waived due to the principle of optional, anonymous, and non-compensated participation. The questionnaire was developed by the authors—obesity experts working in primary care, medical specialties (diabetology, endocrinology), and psychology. It comprised 31 questions, which included single or multiple choice, numeric response, and ranking formats. The questionnaire was divided into four main sections: demographic data (area of expertise, gender, age group, and BMI), professional views on obesity and obesity management, personal perspectives on the subject (unconscious bias), and practical management approaches (see Supplementary Tables S1–S4). Some questions were taken from the ACTION-IO study [7] to enable direct data comparison. The estimated time to complete the questionnaire was 5 min, and 47 variables were collected. To ensure clarity, timing, data quality, and relevance to the study objectives, pre-testing was conducted with 10 participants from both medical and non-medical backgrounds.

The link to the online questionnaire was then distributed via e-mail to 11,597 members of the Medical Chamber of Slovenia—the Slovenian physician registry, enabling the researchers to maintain a neutral approach. Participation in the study was optional, anonymous, and non-compensated. The online questionnaire was active for two weeks (19 February–5 March 2024). The target sample size was 10% of the physician registry. The outcomes were measured using single and multiple item selection, numeric response, and ranking.

Finally, a combination of questions was used to estimate the individual’s professional and personal (unconscious bias) perspectives on obesity. Factor analysis was used to confirm the validity of these question constructs. Every answer was scored using a scoring system shown in the appendix (Table A1 and Table A2). Hence, construct scores ranged 0–4 pts for the professional and 0–5 pts for the personal perspective. In both cases, a higher score indicates a better understanding of the biological and pathophysiological basis of obesity, as well as a less stigmatizing and less judgmental attitude.

### 2.2. Statistical Analysis

Statistical analysis was conducted using SPSS (IBM, version 23.0, Armonk, NY, USA) and the R programming language (R Core Team, version 4.4.2, 2021, Vienna, Austria). Descriptive statistics such as mean ± standard deviation (SD), median (Q1–Q3), and frequency (%) were utilized to summarize the data. Normality tests including the Shapiro–Wilk and Kolmogorov–Smirnov tests were performed.

To compare the responses across different healthcare professionals (HCPs), genders, and among age and BMI groups, Pearson’s Chi-squared test or Fisher’s Exact test was employed. The Mann–Whitney U test was used to compare the overall professional and personal perspectives on obesity between genders. The Kruskal–Wallis rank sum test was applied to compare these perspectives across different HCPs, age groups, and BMI groups. For post hoc analysis, the Dunn test with Bonferroni correction was applied to adjust for multiple comparisons.

Statistical significance was set at *p* < 0.05, using two-tailed tests. Only data from participants who completed the survey were included in the analyses.

## 3. Results

A total of 1248 physicians opened the survey link, representing a 10.8% response rate to the Medical Chamber’s invitation. Of these, 898 (72%) engaged with the survey, with 789 (63.7%) fully completing the questionnaire, and 103 providing partial responses. The average completion time was 3 min and 43 s.

### 3.1. Demographic Data

Responders were predominately women (73.7%, n = 577), age 30–40 (27.2%; n = 214), and with a BMI < 25 kg/m^2^ (56.8%: n = 448). The distribution in other age groups was as follows: 7.5% (n = 59) of responders were under 30 years old, 22.2% (n = 175) aged 41–50 years, 18.5% (n = 146) aged 51–60, and 24.6% (n = 194) were over 60 years of age. According to their BMI, 30.5% (n = 241) of responders were overweight (BMI 25–30 kg/m^2^), 8.4% (n = 66) live with obesity class I (BMI 31–35 kg/m^2^), 1.1% (n = 9) with obesity class II (BMI 36–40 kg/m^2^), and 0.6% (n = 5) with obesity class III (BMI > 40 kg/m^2^). Furthermore, 2.5% (n = 20) of responders decided not to give information on their BMI. According to their areas of expertise, 16.6% (n = 131) of the HCPs were (junior) resident physicians, 14.7% (n = 116) primary care physicians (PCPs), 11.5% (n = 91) internal medicine specialists (and/or subspecialists), 8.4% (n = 66) surgeons (and/or surgical subspecialists), 17.3% (n = 136) dentists, and 31.5% (n = 248) HCPs of other specialisms.

### 3.2. Professional Standpoint on Obesity and Obesity Management

In general, 93.6% (n = 733) of physicians agree that obesity is a disease and 88.4% (n = 688) see it as equally important as other diseases. Over three-quarters (75.5%; n = 593) of physicians are professionally interested in the subject, females more than males (*p* < 0.001), and interest grows with physicians’ age (*p* = 0.006). The majority (83.7%; n = 652) are familiar with the definition of obesity, but only 43.1% (n = 333) know The International Classification of Diseases (ICD) obesity code and even fewer (39%; n = 300) use it. Coding grows with physicians’ age (*p* = 0.002). Of those not coding obesity (61.0%; n = 469), most do not use the ICD coding system at all (80.3%; n = 374) or code the main visit problem only (44.6%; n = 208). Less often, no influence on the billing (17%; n = 79) or something else (26.6%; n = 124) were used to explain non-coding, but the least selected reasons were not finding obesity important regarding the visit problem (15.2%; n = 71) and not finding obesity important as a disease in general (1.7%; n = 8).

**Figure 1:**
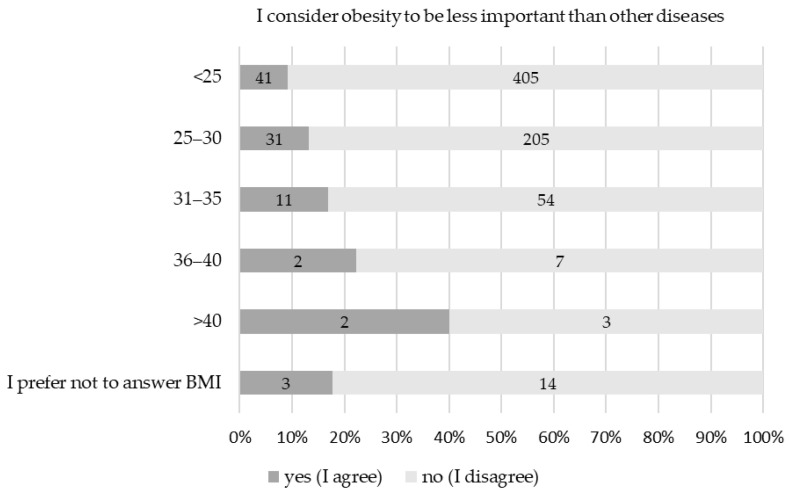
Physicians with higher BMI find obesity less important than other diseases.

There were, however, significant differences between HCPs’ areas of expertise: surgeons and dentists were less interested in the subject (*p* = 0.001), less often considered obesity as a disease (*p* = 0.008), less often knew the obesity definition (*p* < 0.001), and less often knew and/or used the ICD obesity code (*p* < 0.001 for both). The opposite goes for PCPs, followed by specialists in internal medicine. Importantly, physicians with a higher BMI also found obesity less important than other diseases (*p* = 0.036), as shown in Figure 1.

Most physicians believe that people living with obesity are treated equally (57.3%; n = 452) or worse (36.8%; n = 290) in the healthcare system. Additionally, 57.9% (n = 453) of physicians do not assume that patients are fully responsible for their weight, with female physicians showing greater leniency (*p* = 0.004). The majority (71.2%; n = 555) of physicians do not feel uncomfortable discussing patients’ weight; however, (junior) resident doctors and dentists are notably less comfortable in addressing this topic (*p* < 0.001). Physician experience, correlated with age, also plays a significant role (*p* < 0.001).

Although 81.3% (n = 631) believe that they could help their patients with losing weight, even more (84.5%; n = 664) feel that their patients would therefore need to completely change their lifestyle. This belief increases with doctors’ age (*p* < 0.001). The majority (82.0%; n = 641) also think that patients could lose weight if a serious attempt were to be made in this direction. Male physicians were especially supportive of this statement (*p* < 0.001) and a significant association was also confirmed with physicians’ age (*p* < 0.001).

Finally, our combined professional standpoint assessment further confirmed a significant difference among different HCP categories (*p* = 0.007), gender (*p* <0.001), and BMI (*p* = 0.003). Surgeons and dentists scored lower (median 1.5 [0.5;2.0] both) in comparison to all other HCP categories (median 1.5 [1.0;2.0] for all; *p* < 0.001). Men scored lower than women (median 1.5 [0.5;2.0] vs. 1.5 [1.0;2.0]; *p* < 0.001).

Although significant differences were confirmed for different BMI groups, there were no clear gradual tendencies in scoring (possibly due to low absolute numbers). There was no difference in age (*p* = 0.197).

### 3.3. Intimate/Personal View on Obesity and Obesity Management

The physician’s intimate/personal view reflecting unconscious bias on obesity being a disease significantly differs from their professional standpoint: only 74.0% (n = 582) feel that obesity is a disease and 26% (n = 205) are convinced on the contrary (*p* < 0.001). There were no significant differences regarding HCPs’ area of expertise, age, gender, or BMI.

Although 46.3% (n = 364) do not demonstrate specific feelings towards obesity, 49.4% (n = 389) associate it with unpleasant feelings (e.g., laziness or lack of willpower) and only 4.3% (n = 34) to pleasant ones (e.g., good spirit or hedonism). The proportion of HCPs with unpleasant feelings towards obesity decreases with a rising BMI: 53.2% (n = 238) in BMI < 25 kg/m^2^, 47.9% (n = 115) in BMI 25–30 kg/m^2^, 37.9% (n = 25) in BMI 31–35 kg/m^2^, 33.3% (n = 3) in BMI 36–40 kg/m^2^, and 20% (n = 1) in BMI > 40 kg/m^2^. Almost one-quarter (24.7%; n = 195) of HCPs admitted to using derogatory obesity phrases in their free time. This was significantly associated with a younger age (*p* < 0.001) and lower BMI (*p* = 0.004).

Nevertheless, 82.5% (n = 650) of physicians feel that people of a normal weight also struggle with their body weight. The majority disagreed when asked if people with overweight or obesity do not care for their weight and health (no for weight: 93.8% (n = 738); no for health: 90.9%; n = 716). There were significant differences regarding gender in these three questions, with male doctors being more judgmental (*p* values 0.002, <0.001, and <0.001, respectively).

Finally, using composite answers, we found that unconscious bias was significantly influenced by gender (*p* = 0.017), age (*p* < 0.001), and BMI (*p* = 0.005), but did not differ between HCP categories (areas of expertise; *p* = 0.067). Men scored lower than women: median 3.0 [2.0;4.0] vs. 3.0 [3.0;4.0] (*p* value = 0.017). Scoring increased with age: median 3.0 [2.0;4.0] in all age groups < 40 years old vs. 3.0 [3.0;4.0] in age groups > 41 years. Scoring increased with BMI: median 3.0 [2.0;4.0] was calculated for BMI < 25 kg/m^2^, 3.0 [3.0;4.0] for BMI 25–30 kg/m^2^, and 4.0 [3.0;4.0] for all other BMI groups.

### 3.4. Practical Management

When considering obesity management, physicians are mostly guided by obesity-related complications (56.7%; n = 446), clinical assessment of nutrition (20.2%; n = 159), and to a lesser extent, by BMI (13.6%; n = 107) or waist circumference (9.4%; n = 74). There were significant gender differences: female doctors were guided more by obesity-related complications and male doctors by BMI (*p* = 0.010).

When asked about obesity complications that are best prevented by losing weight, physicians most commonly recognized diabetes (95.7%; n = 754), cardio-vascular events (90.9%; n = 716), arterial hypertension (86.3%; n = 680), obstructive sleep apnea syndrome (80.2%; n = 632), and hyperlipidemia (74.9%; n = 590). Depression and osteoarthritis were recognized less often (53.7% (n = 423) and 51.5% (n = 406), respectively). Female doctors chose depression more often (*p* = 0.001), and obstructive sleep apnea syndrome was less often chosen by dentists and surgeons (*p* < 0.001).

Weight loss of 5–15% was mostly (63.4%; n = 486) considered successful for preventing obesity complications/comorbidities and >15% weight loss in one-third (33.8%; n = 259) of responders. No differences regarding the demographic data were noted.

**Figure 2:**
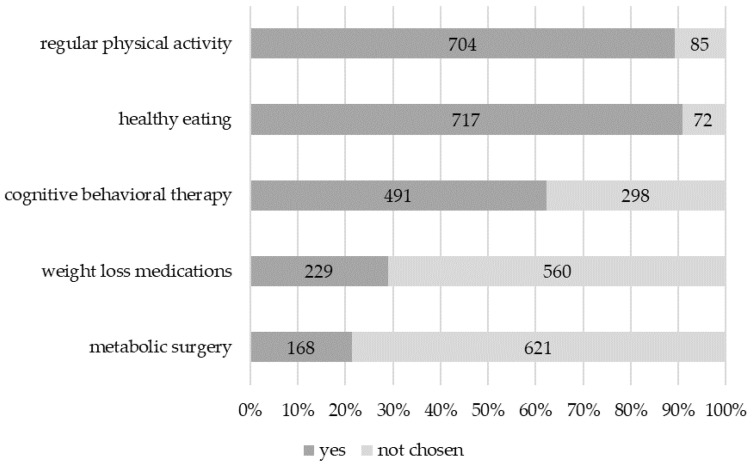
Perceptions of the most effective weight loss method.

When asked about the most effective weight loss method, physicians still most frequently chose healthy eating (90.9%; n = 717) and regular physical activity (89.2%; n = 704), whereas cognitive behavioral therapy (CBT), weight loss medications, and metabolic surgery were less frequently chosen (Figure 2). CBT was more often chosen by female doctors (*p* < 0.001) and weight loss medications were more often chosen by PCPs and internal medicine specialists (*p* < 0.001).

Most physicians referred their patients to the local Health Promotion Center (51.3%; n = 403), one-quarter referred them to metabolic surgery (25%; n = 196), and 20% (n = 162) at some point prescribed weight loss medications. In general, physicians are not familiar with the Slovenian guidelines on weight loss medications (63.4%; n = 498). This knowledge is higher in PCPs and internal medicine specialists (*p* < 0.001) and increases with age (*p* < 0.001). Most also feel that the elderly should be treated differently (53.6%; n = 418), with significant differences, with PCPs and internal medicine specialists choosing this option most often and dentists the least (*p* < 0.001).

## 4. Discussion

Firstly, our demographic data match the national findings regarding obesity prevalence in Slovenia, as only 10.4% of our highly educated participants (of those willing to share their BMI) meet the obesity BMI criteria [2].

Secondly, our results show the need for education on new insights into obesity pathophysiology, shifting obesity perceptions from seeing it as a risk factor to acknowledging obesity as a serious chronic disease. Upon first glance, the idea of obesity being a disease (as opposed to being a risk factor only) is highly accepted in our professional community, with 93.6% of our responders supporting this statement. In comparison, 88% of HCPs in ACTION-IO agreed that obesity is a chronic disease [7]. The majority (71.2%) do not feel uncomfortable talking about patient’s weight and over one-third (36.8%) acknowledge that people living with obesity are treated worse in the healthcare system.

However, there is a significant misalignment between professional standpoints and unconscious bias towards obesity, with only 74.0% intimate/personal confirmations of obesity being a disease. No differences in participant demographic data regarding this statement suggest that it reflects (unaware?) weight-related stigma. As most communication is non-verbal [9], the divergence of beliefs due to stigma presents potential for judgmental, stigmatizing, non-verbal expressions in clinical settings. Furthermore, almost half (49.4%) of our responders associate obesity with unpleasant feelings (e.g., laziness or lack of willpower) and almost one-quarter (24.7%) admitted to using derogatory obesity phrases in their free time. All this represents a realistic foundation for possible bias in further care delivery. Strong implicit and explicit anti-fat bias was already previously described as widespread among medical doctors [10]. Studies suggest that 29.9% of healthcare professionals perceive obesity as shameful [11], and individuals, including medical doctors, may feel that it is socially acceptable to express negative attitudes about overweight people [10].

On the other hand, the misalignment between professional standpoints and unconscious bias towards obesity can also be related to an inability to internalize a belief that is in contrast to the acknowledged simplistic obesity concept [12]. This would explain the divergence between the argued standpoint and simultaneously insisting on common misbeliefs, such as the most effective weight loss methods being healthy eating (90.9%) and regular physical activity (89.2%), the need for a complete lifestyle change (84.5%), or a lack of serious motivation to lose weight (82.0%). Regardless of the underlying reason, these beliefs contribute to ineffective obesity management, even more so because 81.3% of HCPs believe that they could help their patients lose weight. Eat less, move more, and use willpower is oversimplified advice on how to lose weight [13].

Looking at composite professional vs. intimate/personal outcomes, physicians’ gender and BMI significantly affected both. As expected, HCPs’ area of expertise impacted physicians’ professional (but not personal) standpoints. Age affected personal beliefs only. Physicians with a higher BMI found obesity to be less important than other diseases (*p* = 0.036), which could also affect their care delivery. This is in accordance with previous findings reporting that physicians without obesity are more likely to document obesity [14], and that physicians with a normal BMI are more likely to engage their patients with obesity in weight loss discussions [15]. This is important since diagnosing obesity can be the first step to weight loss [16]. On the other hand, physicians with lower BMIs scored lower in our composite answers, reflecting a more judgmental unconscious bias. This suggestion is indirectly supported by the inverse relationship between unpleasant feelings towards obesity and physicians’ BMI. A lower BMI was also significantly (*p* = 0.004) associated with responders using derogatory obesity phrases in their free time.

In addition to BMI, we also confirmed gender differences, suggesting the influence of gender on both professional attitudes and unconscious bias towards obesity. Similarly, previous studies consistently showed a lower weight bias in women [10,11]. We found female physicians to be less judgmental; fewer assumed patients to be fully responsible for their weight (*p* = 0.004), fewer assumed that patients with overweight/obesity do not care about their weight (*p* < 0.0001) nor health (*p* < 0.001), and fewer believed that patients could lose weight if a serious attempt were to be made (*p* < 0.001).

Based on our findings, we offer insights into possible changes to the educational approach toward overweight and obesity according to gender and age differences, and also areas of expertise. Our gender-related differences suggest that male physicians seem to rely more on tangible numerical data (e.g., they significantly more often use BMI when deciding on obesity treatment) and consider psychological aspects less often (e.g., recognizing depression, considering the use of CBT, and approaches towards the elderly with obesity). Currently, the simplistic obesity paradigm is based on the false assumption that individuals can fully control their body weight through appropriate behavioral choices, and that obesity is a self-induced, easily reversible condition [12]. Presenting correlations with other clinical conditions and outcomes can further fortify obesity as a risk factor rather than an independent chronic disease. We believe that it is important to clearly present obesity as a »neuroendocrine disease« with endocrine and neural mechanisms of weight control and energy homeostasis [17] and advances from genetic technology [18]. Tools to visually present the complexity of and possible interactions leading to obesity development (e.g., Foresight obesity system map [19]) should also be used. Such an in-depth understanding could also help younger physicians, who are currently less comfortable addressing the problem of obesity and lack knowledge of the coding and guidelines on weight loss medications. Dentists are less comfortable talking about obesity, and more often feel they cannot help people with obesity. In dentistry, obesity is recognized as a growing problem, as awareness on how it affects access to dental services and dental management is spreading [20]. Dentists emphasize a lack of knowledge or protocols for managing the clinical and practical implications of obesity [20].

Studies suggest that HCPs should strategically focus on reducing obesity bias and provide high-quality obesity management [8]. Distributing a nationwide survey through the highest national professional body helps to build obesity awareness and gain insight into common misconceptions. Furthermore, our long-term goal is to prevent physicians from giving oversimplified advice on weight management (eat less, move more) and to encourage them to address the issue with the patient in a proper way. Therefore, our questionnaire included self-reflective items prompting participants to evaluate their attitudes toward obesity. Respondents were asked to indicate whether they associated obesity with negative stereotypes (e.g., laziness or lack of willpower), whether they used derogatory language when discussing obesity outside of professional settings, and whether they believed that people with obesity sufficiently cared about their health. Additionally, an open-ended reflection question invited participants to share insights on how their perceptions of obesity may influence their clinical practice. Responses were analyzed qualitatively to identify recurring themes related to self-awareness, cognitive dissonance, and shifts in perspective. Also, our research unobtrusively encouraged HCPs to gain knowledge on different treatment possibilities. In the era of constant information accessibility, people living with obesity are eagerly expecting new treatment possibilities. We were surprised by the data regarding practical obesity treatment, as more physicians referred their patients to metabolic surgery (25%) than prescribed them with weight loss medications at some point (20%). This is partially due to the easy accessibility of metabolic surgery and the lack of knowledge on the national guidelines on weight loss medications [21], but mostly due to the (un)availability of weight loss medications in our country. When talking about health inequities, our awareness is focused on vulnerable populations (people with disabilities or marginalized racial and ethnic groups, etc.) [22]; however, in comparison to diabetes and other chronic diseases, people with obesity are placed in an extremely subordinate position regarding access to and quality of care.

Our study has some limitations. It was conducted online, potentially excluding individuals with a lack of computer skills or internet access. In theory, the possibility of repeated survey entries also existed (unlikely). The data are self-reported, which may introduce response bias, especially social desirability bias. An attempt to reduce this risk was made by separating the sets of questions regarding professional/personal attitudes and including self-reflective items, offering participants the opportunity to delineate their standpoints, and thus, the awareness of such differences are not only possible, but probable. However, further work is needed to compare these findings with international data and explore intervention strategies to mitigate bias. Through feedback upon study completion, participants suggested additional HCP subcategories to help improve obesity care in target groups (e.g., pediatricians, bariatric surgeons, and psychiatrists). Additional data on actual clinical work and/or years of clinical practice would also be beneficial to enhance the validity of the results.

## 5. Conclusions

Overall, distributing a nationwide survey on obesity through the highest national professional body helps to build obesity awareness and gain insight into common misconceptions. HCPs’ area of expertise impacted their professional standpoint (suggesting conscious bias), whereas male gender, the younger age, and the lower BMI of HCPs affected unconscious bias toward obesity and its effective care. The findings can help to individualize educational strategies and create a more equitable environment in obesity healthcare.

## Data Availability

The data presented in this study are available upon request from the corresponding author due to privacy.

## Supplementary Materials

The following supporting information can be downloaded at: https://www.mdpi.com/article/10.3390/jcm14051486/s1, Obesity—Attitudes of Slovenian Doctors and Dentists (questionnaire): Table S1: Section 1—Basic demographic information; Table S2: Section 2—What is your PROFESSIONAL stance?; Table S3: Section 3—What is your PERSONAL attitude toward obesity?; Table S4: Section 4—Treatment.

## Abbreviations

The following abbreviations are used in this manuscript:

HCP	Healthcare professional
ICD	International Statistical Classification of Diseases and Related Health Problems
BMI	Body mass index
SD	Standard deviation
PCP	Primary care physicians
CBT	Cognitive behavioral therapy

## Appendix A

**Table A1:** Assessment of professional perspectives on obesity using four questions.

Statement	Scoring (pts)		
Patients are fully responsible for their weight.	yes = 0		no = 1
To lose weight, patients would have to completely change their lifestyle.	yes = 0		no = 1
Patients could lose weight if they were to make a serious attempt.	yes = 0		no = 1
Within the healthcare system, people with overweight or obesity receive a (better/same/worse) treatment.	better = 0	same = 0.5	worse = 1
Ʃ	0–4		

**Table A2:** Assessment of unconscious bias on obesity using four questions.

Statement	Scoring (pts)		
When not at work, I find myself using derogatory obesity phrases.	yes = 0	no = 1	
People with overweight or obesity do not care for their weight.	yes = 0	no = 1	
People with overweight or obesity do not care for their health.	yes = 0	no = 1	
I relate obesity to pleasant (e.g., good spirit or hedonism)/not extra/unpleasant (e.g., laziness or lack of willpower) feelings.	unpleasant = 0	not extra = 1	pleasant = 2
Ʃ	0–5		
