# Supplementary material for: Factors Affecting the Unconscious Bias of Healthcare Professionals in Obesity Care
Source: J Clin Med. 2025 Feb 23;14(5):1486. doi: 10.3390/jcm14051486 (PMC11900521; doi:10.3390/jcm14051486)
Supplement: Supplementary file 1 [file jcm-14-01486-s001.zip › jcm-3484443-supplementary.pdf]

Supplementary Materials: Obesity – Attitudes of Slovenian Doctors and Dentists (questionnaire)

Table S1. Section 1—Basic demographic information

No.	question	answer
Q1	**Your professional specialization:**	• (junior) resident physician<br>• primary care physician (PCP) / general practitioner<br>• internal medicine specialist (or an internal medicine subspecialty)<br>• surgeon (and/or surgical subspecialty)<br>• dentist<br>• other
Q2	**Your gender:**	• male<br>• female
Q3	**Your age:**	• <30 years<br>• 30-40 years<br>• 41-50 years<br>• 51-60 years<br>• > 60 year
Q4	**Your body mass index (BMI)** (kg/m^2):	• < 25<br>• 25-30<br>• 31-35<br>• 36-40<br>• >40<br>• I prefer not to answer

Table S2. Section 2—What is your PROFESSIONAL stance?

No.	statement			
Q5	**As a healthcare professional, I believe/assess/agree…**	obesity is a disease	yes	no
Q6		I am familiar with the definition of obesity	yes	no
Q7		I know the obesity code in the ICD system	yes	no
Q8		I code the diagnosis of “obesity” during patient assessment	yes	no
*Q8a	***If you do NOT code the diagnosis of “obesity,” why not?** (multiple choice)	I do not use the ICD coding system<br>I only code the main diagnosis or reason for the visit<br>I do not consider the diagnosis important for my evaluation<br>I generally do not consider “obesity” to be an important diagnosis<br>It does not affect the billing of my services<br>Other		
Q9	**Your attitude towards patients living with overweight or obesity:**	A patient’s body weight is entirely their own responsibility†	yes	no
Q10		To lose weight, patients must completely change their lifestyle†	yes	no
Q11		Patients could lose weight if they really set their mind to it†	yes	no
Q12		I feel uncomfortable discussing my patients’ weight unless they mention it first†	yes	no
Q13		I consider obesity to be less important than other diseases†	yes	no
Q14		I cannot help patients lose weight	yes	no
Q15	**I am interested in the field of obesity professionally**		yes	no
Q16	**I believe that people with overweight or obesity receive:**	• the same treatment as everyone else<br>• worse treatment<br>• better treatment		

†question from or similar to the ACTION-IO study

Table S3. Section 3—What is your PERSONAL attitude toward obesity?

No.	statement		
**Q17**	**I associate obesity with:**	• positive feelings (e.g., good mood, hedonism)<br>• negative feelings (e.g., laziness, lack of willpower)<br>• no particular feelings	
**Q18**	**I personally think of obesity as a disease**	yes	no
**Q19**	**Outside of work, I catch myself using derogatory terms related to obesity in everyday conversations**	yes	no
**Q20**	**Normally nourished individuals do not worry about their body weight**	yes	no
**Q21**	**People with overweight or obesity do not care about their body weight**	yes	no
**Q22**	**People with overweight or obesity do not care about their health**	yes	no

Table S4. Section 4—Treatment

No.	statement		
**Q23**	**When planning obesity treatment, the most important factor for me is:**	• body mass index<br>• waist circumference<br>• clinical assessment of nutritional status<br>• obesity-related complications	
**Q24**	**Which complications do you think are best prevented by weight reduction?**<br>(multiple choice)	• diabetes<br>• arterial hypertension<br>• hyperlipidemia<br>• cardiovascular events<br>• osteoarthritis<br>• depression<br>• sleep apnea syndrome	
**Q25**	**What weight loss percentage do you consider successful for preventing or treating complications and associated diseases?**	• <5%<br>• 5-15%<br>• >15%	
**Q26**	**The most effective therapeutic approach for treating obesity is:**<br>(multiple choice)	• regular physical activity<br>• healthy nutrition<br>• behavioral cognitive therapy<br>• pharmacotherapy<br>• metabolic surgery	
**Q27**	**In my clinical practice, I have recommended treatment at a health promotion center for a person with obesity**	yes	no
**Q28**	**I am familiar with Slovenian guidelines for treating obesity with medications**	yes	no
**Q29**	**In my clinical practice, I have prescribed weight-loss medications**	yes	no
***Q29a**	***If YES, you have prescribed weight-loss medications, how often?**<br>• yes, frequently<br>• yes, occasionally<br>• yes, but only rarely		
**Q30**	**In my clinical practice, I have referred a person with obesity to metabolic surgery**	yes	no
**Q31**	**Is your approach to managing obesity in elderly patients different?**	yes	no
